# Being fast or cautious? Sociocultural conditions influencing the sexual pathways of Black females in the United States

**DOI:** 10.1186/s12905-022-01644-x

**Published:** 2022-03-13

**Authors:** Natasha Crooks, Barbara King, Audrey Tluczek

**Affiliations:** 1 Department of Human Development Nursing Science, College of Nursing, University of Illinois Chicago, 845 S. Damen Avenue Room 816, Chicago, IL 60612 USA; 2 School of Nursing, University of Wisconsin-Madison, Madison, WI USA

**Keywords:** Sexual health, Black, Grounded theory, Women’s health, Sexually transmitted infections, Theory development, Qualitative research

## Abstract

**Background:**

Black females in the United States face unique sociocultural conditions that impact their sexual development and increase their risk for sexually transmitted infections (STI), including but not limited to chlamydia, gonorrhea, and HIV. Research has not adequately explained how sociocultural conditions contribute to this increased risk. The purpose of our investigation was to explore the sociocultural conditions that influence Black cisgender females risk for STI.

**Methods:**

This grounded theory study involved in-depth audio-recorded interviews with 20, primarily heterosexual, Black females ages 19–62.

**Results:**

Findings informed a conceptual model that builds on previous theory about the sexual development of Black females and explains how sociocultural conditions impact two, participant identified, sexual pathways: Fast and Cautious. Movement on these sexual pathways was not always a linear trajectory; some participants shifted between pathways as their sociocultural contexts changed (i.e., sexual assault, STI, and level of protection). The Fast sexual pathway often led to greater STI risk.

**Conclusions:**

This model may inform future research designed to prevent STI/HIV and promote the sexual health of Black females across the life course.

## Background

Black females experience significant sexual health disparities and sexual victimization in the United States (US) [1]. About 20% of Black women (> 23 million) are raped during their lifetimes [2, 3]. Black sexual minority women experience the highest rates of physical and sexual victimization in adolescence and adulthood among women overall [3, 4]. Black women have the highest rates of sexually transmitted infections (STI) despite having fewer partners, higher condom use, and engaging in less risky sex than white or Hispanic women [1, 5]. Of all women diagnosed with Human Immunodeficiency Virus (HIV) in 2018, about 57% were Black [6]. The rate of chlamydia infection among Black girls aged 15–19 years (6771.6 cases per 100,000 girls) is 4.5 times the rate among white girls in the same age group (1518.5 cases per 100,000 girls) [1, 6]. Black girls are more than twice as likely as their white peers to become pregnant [7]. A history of sexual trauma can also affect Black female sexual behavior in ways that place them at greater risk for STI [3, 8, 9]. The aforementioned research has primarily focused on the experiences of STI among Black cisgender females. This paper focuses on the sexual experiences of Black girls and women in the US.

Researchers have identified sociocultural conditions (e.g., sexualized stereotypes, gender, and race-based discrimination, and cultural norms) unique to heterosexual Black girls and women that contribute to sexual behaviors which place them at risk for STI [10–15]. Black culture, in the context of this study, refers to the collective history, values, customs, world views, linguistic meanings, and messages shared by those who identify as being Black in America [16]. For example, Black cultural messages such as “fast” or “fast tailed girl” contribute to the sexual socialization and behaviors of Black girls and women [11, 17, 18]. This cultural message/label creates shame in sexual exploration and silences Black girls in regards to communication about sexual health, consequentially increasing STI risk [11, 17, 18]. Being “fast” or the label of “fast tailed girl” coincides with the hypersexual Jezebel stereotype perpetuated by racist policies (i.e., forced sterilization of Black women) made to control Black female sexual and reproductive health [19, 20]. A survey of 249 Black women showed that women aged 18–34 were more likely to endorse the Jezebel stereotype than Black women 55 years and older [21]. Although previous researchers acknowledge that Black girls and women face unique sociocultural challenges, to date, there is one conceptual model that explores how such conditions contribute to their sexual development and STI risk [11]. This study extends that work, by developing a conceptual framework to explain the sociocultural conditions that influence Black female sexual development, behavioral pathways, and STI risk.

### Sociocultural conditions influencing Black female sexuality

Several sociocultural conditions have been found to influence Black female sexuality. Many Black females suffer psychological trauma due to a combination of race and gender-based discrimination or misogynoir that can lead to the internalization of negative societal messages of being devalued which adversely affect their views of themselves [13, 22]. Misogynoir refers to the intersecting forms of oppression (sexism and racism) that Black women face, often leading to the erasure of their experience [23]. For example, many Black women describe experiencing hatred, dislike, distrust, and prejudice directed towards them simply because of their identity, leading to a lack of compassion or disbelief of their stories and experiences, more specifically stories of sexual assault [23]. However, researchers have identified individual factors (i.e., high self-esteem, racial-ethnic esteem, and self-image) that serve to protect Black females from adverse mental health outcomes associated with racism and discrimination [20, 21, 24]. Findings from a survey of 262 middle-class Black women, aged 18–78, suggested certain paternal cultural practices (i.e., cultural embeddedness and ethnic pride) were negatively related to communication about their partner’s sexual history; body esteem was positively associated with inquiring about their partner’s previous sexual history [25]. Still, there remains concern about the negative impact of internalized societal messages on Black female sexuality. Several researchers found that perceptions of low social status can affect female behavior across many social contexts including sexual encounters [10, 14, 25, 26]. However, sexuality and sexual behaviors are influenced by cultural norms dictated by race and gender [13, 27]. There is minimal research that explores the intersection of race, gender, and sexuality and their influence on Black female sexual development, which is essential to addressing sexual health disparities within this population [22].

Researchers have suggested that the American legal system, in addition to disproportionate levels of unemployment, poverty, and mortality, impacts Black communities and heterosexual sexual networks in several damaging ways [10, 28, 29]. Discriminatory legal practices (e.g., racial profiling, disproportionately high conviction rates, and prolonged sentences) result in the mass incarceration of Black men. Removal of these men from their communities disrupts Black family units and deprives Black girls of their fathers or father figures whose social roles include protecting their daughters from sexual predation [17, 30]. With so many Black men imprisoned or dying due to homicide or infant mortality, the pool of potential Black male sexual partners is drastically reduced [10, 28]. Thus, sexually active heterosexual Black females in the US may be more likely than their white counterparts to have overlapping sexual relationships, also known as sexual concurrency, which increases their risk for STI and HIV [10, 15]. Black heterosexual populations are more likely to select sexual partners with similar characteristics as their partners, such as race. This phenomenon is termed assortative mixing and can increase concentrations of STI among Black sexual networks [31]. In a quantitative study with 570 Black adolescent girls aged 15–21, participants identified relationship factors (i.e., partner age and future of relationship) as important contributors to the prevalence of sexual concurrency among Black girls and women [15]. Internationally, a qualitative study with 37 Black girls and boys 15–17 years old also identified concurrent sexual partnerships, mismatched perceptions and expectations, and barriers to condom use contributing to STI risk. Furthermore, the racialized nature of American society tends to segregate sexual coupling. The context of Black heterosexual relationships leaves Black girls and women vulnerable to STI [30].

Additionally, Black girls in the US experience puberty at younger ages than girls of other ethnicities, which has been associated with early sexual activity, engagement in risky sexual behaviors and increased STI risk [32–34]. Early sexual engagement is defined as having sex under the age of 16 years old [35]. Some Black women have reported that as young girls, they experienced early pubertal onset and older men often treated them like women, pursuing them sexually [30]. Furthermore, researchers found that stereotyped sexual messaging about Black females in the popular media (i.e., music, movies, and television) can prompt Black girls to mimic such sexualized behaviors and act older than their chronological ages [12, 17, 36, 37]. Black feminist scholars have contended that historical stereotyped racial images (i.e., Jezebel, Mammy and Sapphire) persist in contemporary society and these images continue to affect how Black girls and women view themselves and are viewed by others [20, 38]. The combination of these sociocultural conditions often leaves Black girls and women systemically unprotected and vulnerable to sexual predation [17]. Therefore, more research is needed to understand how these conditions influence Black female sexual development.

### Life course perspective to understand Black female sexuality

From early childhood and throughout the life course, sexuality is shaped by psychological, social, and cultural contexts. Therefore, it is important to examine these factors in the study of female sexuality [39]. Research informed by a life course perspective allows researchers to examine the interactions of multiple sociocultural conditions as well as their cumulative influence on health and across generations of Black girls and women [39]. Additionally, it is important to understand the behaviors that occur during various developmental stages and how events and experiences affect later stages of development. Although prior literature has identified sociocultural conditions that influence STI risk [10–15], researchers have not explored the subjective perspectives of Black girls and women about how these conditions affect their sexual development across the life course.

There is a large gap in our understanding of Black female sexual development and behavioral pathways as much of the literature has focused on sexual risk. Previous research about female sexual development has been predominantly based on white females, with little attention given to Black female experiences [40]. A content analysis of sexuality research in counseling psychology suggests that when such research is conducted with people of color, the discourse is sex-negative [40]. Most research about Black female development has failed to incorporate culture and intersecting identities [41–43]. A more comprehensive understanding of female sexual development is needed to discern how Black girls enter womanhood, particularly regarding their sexuality.

To the best of our knowledge, only one theoretical model of Black female sexual development has been published [11]. This theoretical model is based on a life course perspective and describes three distinct sequential developmental phases (i.e., Girl, Grown and Woman) of becoming a Black woman [11]*.* Participants’ sexual experiences during the Girl phase (ages 5–14 years) were marked by naivety, vulnerability, and lack of control over their bodies. The Grown^1^ phase may start as early as 11 years old and was characterized as a confusing time during which participants were “figuring out” their sexual identities. The Woman phase (ages ≥ 18 years) was often associated with becoming a parent and a period of new insights, personal growth, and emotional strength [11]. This process of figuring out sexual identities doesn’t necessarily stop at the Grown phase, but is ongoing throughout their life course.

The purpose of our investigation was to explore the sociocultural conditions that influence Black cisgender females’ risk for STI. This article builds on previous work to provide an expanded conceptualization of Black female sexuality. Grounded theory was chosen for this study as it is based on the social psychology theory of symbolic interactionism which facilitates investigation of social processes associated with phenomena that lack adequate theoretical explanation [44, 45]. Given the lack of knowledge about Black female sexual development, particularly from their perspectives [46], grounded theory was well-suited for this study.

## Method

### Study population, participants and sampling

Purposive and theoretical sampling guided recruitment through flyers and word of mouth in university and community settings. We initially used purposive sampling to recruit females aged 18–24 who self-identified as Black and had a history of STI. We wanted to gain insight about sociocultural conditions that might explain this increased risk [47]. Early data analysis of 18–24-year-olds suggested that older Black women might have some influence on Black girls’ sexuality. Therefore, after the first three interviews, we made a theoretical sampling decision to expand recruitment to identify and recruit individuals who could help us achieve a richer understanding of how sociocultural conditions influence the process of becoming a sexual Black woman. The “classification of Black” is defined as anyone having African ancestry, including those of mixed race and Black immigrants to the US [48]. However, given that we drew from a social constructivist perspective [49], we included participants who self-reported their identities as Black. Our inclusion criteria required participants to: (a) self-identify as Black and female, (b) be ≥ 18 years of age, and (c) be fluent in speaking and reading English. Sampling continued until saturation was reached when no new properties, dimensions, or conditions were identified in the analysis [50]. Saturation occurred after interviews with 20 participants [51].

### Procedures

The first author conducted all in-person interviews. To assure protection of participants’ privacy and confidentiality, consent was only obtained verbally. The consent process occurred prior to conducting interviews for data collection. Participants were informed that their participation was voluntary and they could withdraw at any time. In-depth, one-on-one, audio-recorded interviews were conducted between May 2016 and January 2017. Each participant was interviewed once; interviews averaged 55 min. Participants were compensated $30. Interviews were conducted at times and in private locations convenient to participants. Interview questions were developed by the research team and adapted using feedback from a community advisory group representative of the sample. Initial interviews began with open-ended questions. For example, we asked participants, “Can you tell me about things in your life that have contributed to your STI diagnosis or risk?” As categories developed and variation in the sampling continued, interview questions became more focused to identify dimensions and conditions related to the emerging conceptual model [50]. Examples of more focused questions included, “Some participants have described being fast or the Fast pathway. Can you describe what this means? What makes someone fast?” Fast was a term introduced by participants. Recognizing the importance of amplifying Black female voices, we incorporated their language into more focused questions. Pseudonyms were created when reporting quotes to protect participants’ anonymity and confidentiality. Recorded interviews were transcribed verbatim and checked for accuracy by the interviewer.

As researchers, it is important to acknowledge our social location and positionality in relation to our participants. The first author is a Black well-educated, heterosexual, American woman who conceptualized this study and conducted the interviews. She was likely viewed by participants as an insider as she shared the same ethnicity, gender, and sexual identity as many of them. She was also from the same city where the interviews took place, which may have allowed participants to openly share their experiences during the interview process [52]. The other two authors are white heterosexual, well-educated, American women, who conduct research with vulnerable and historically marginalized populations (i.e., older adults and Native American populations). Their outsider perspectives facilitated bias checks when interpreting data [52].

### Data analytic strategy

The three-member analysis team consisted of experts in grounded theory, adolescent development, and women’s sexual health. The team met weekly throughout the study to discuss coding. Findings were verified by referring back to the original transcripts. Data collection and analysis occurred iteratively using constant comparison with open, axial, and selective coding [45, 50]. Initially, line by line analysis of each transcript was conducted using open coding. This procedure involved assigning labels to portions of transcribed data that reflected analysts’ interpretations. Codes with similar meaning were grouped into categories [50]. Axial coding was then used to identify the relationships between categories [53] and conditions that explained the “when, where, who, how and under what circumstances” [50]. The next step involved selective coding consisted of re-examining data for evidence that might confirm or disconfirm previously identified conceptual relationships (i.e., sexual pathways), deepen understanding of contextual factors (i.e., sociocultural conditions), identification of a core social process (i.e., becoming a Black sexual woman), and extrapolation of salient exemplars (i.e., participant quotes). The final step involved integrating the categories, relationships, and conditions into a conceptual model describing the sociocultural conditions that influence Black female sexual development [50].

To assure methodological rigor, accuracy, credibility, and to minimize subjective bias in data analysis, we used bracketing, memo writing, and member checking [53]. The research team bracketed their assumptions by writing field notes and writing memos about study findings to avoid making judgments that might bias data analysis [54]. Team members challenged each other to show how their interpretations were grounded in the data (i.e., participants’ words) rather than their own subjective projections. Member checking involved discussing parts of analyses with participants to ensure the accuracy and credibility of findings [53]. Based on the data analysis of the first three interviews, we formulated a preliminary conceptual model. Thereafter, once the interview was completed, each participant was asked to assist us with member checking the evolving model. Specifically, participants were shown an illustration of the conceptual model, given a brief explanation about it, and encouraged to provide us feedback about how well it represented their experiences. Modifications were made accordingly. Memos documented methodological decisions made throughout the study. Quotes from multiple participants were used to assure the trustworthiness of concepts.

## Results

### Participants

Participants included 20 Black cisgender females aged 19–62 years (mean 31 years) and their social roles included student, daughter, and/or mother. Of the 20 participants, 16 were recruited from the community and four were from a university campus. Although all participants self-identified as Black, three identified as biracial (i.e., Black and white) and two as Black Latinas. Seven participants reported having children, possessing an average of three children. Sixteen participants identified as single, two as married, and two as divorced. Levels of education varied. One was in high school, nine had high school or Graduate Equivalency Degree (GED)^2^, two had two-year associate/technical college degrees, four had bachelor’s degrees, and three had doctoral degrees. In terms of their current socioeconomic status, 17 participants identified as lower to working class and three middle to upper class. All self-identified as sexually active with a median age of 14 years for first intercourse and an average of 11 years old (range 5–20). Most identified as heterosexual; one as bisexual; two “experimented” sexually with females. The median lifetime number of sexual partners was 10 (range 2–33). Fifteen reported having a history of STI. Six reported having had multiple STI, the most common being chlamydia.

**Fig. 1:**
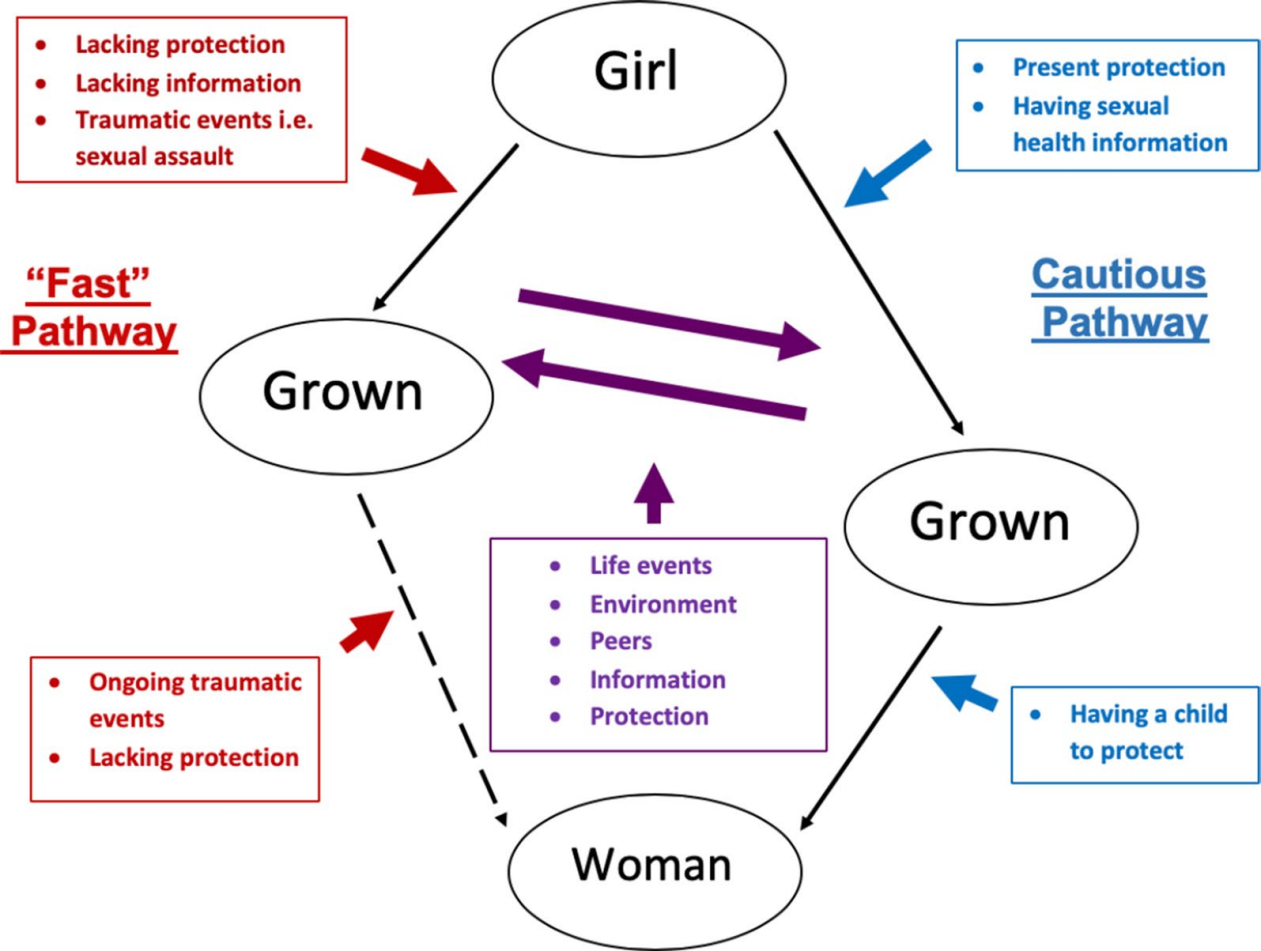
Sociocultural conditions influencing the sexual pathways of Black females

This analysis expands the conceptual model of Black female sexual development across the life course [11]. While the previous model identified three phases of sexual development: Girl, Grown and Woman [11], in this report, two distinct sexual pathways (i.e., Fast versus Cautious) and related sociocultural conditions are described. Sexual pathways were identified and conceptualized as participants’ journeys throughout their sexual development. The Fast pathway was described as an accelerated passage to first sexual encounter, typically at young ages and often led to engaging in high-risk sexual behaviors (i.e., unprotected sex, sex with multiple partners, and STI). The Cautious pathway was characterized by the onset of sexual activity at older ages and safe sexual behaviors. As illustrated in Fig. 1, the conceptual model includes three phases of sexual development (i.e., Girl, Grown, Woman), two pathways (i.e., Fast and Cautious), and sociocultural conditions that influence sexual pathway choices. Participants often described their entrance onto particular pathways as “choices” during all three phases. Although there were circumstances (i.e., sexual trauma or rape) that were not their choice, the pathways were conceptualized as choices based on participants’ reports as they retrospectively recalled their experiences. The dotted line on the Fast pathway represents the uncertainty that some participants expressed about becoming a “woman”. Being a woman was characterized as achieving goals that could be academic (i.e., graduating from college), career (i.e., obtaining jobs in their desired profession), or personal (i.e., having children) as well as having complete control over their sexual identities and behaviors. We also recognize that pleasure is an important aspect of sexual health [55]. However, the grounded theory method used for this study relies on participants taking the lead in identifying what they believe to be most salient to the topic (i.e., STI risk). They emphasized social relationships, rather than the pleasure associated with sex. Perhaps, they focused more on the negative aspects of sex because the purpose of our study was to gain an understanding of sociocultural conditions influencing STI risk, which was perceived as an adverse experience.

### Fast pathway

The Fast pathway was characterized as having early sexual contact, multiple sexual partners, unprotected sex, and sexualized behaviors (i.e., dressing or acting sexual) creating greater sexual risk for Black females. Having sex at a young age was a consequence of entering the Fast pathway. By the time they were "grown” and already on the pathway, being “fast” became a retrospective characteristic. We recognize that using “fast” to describe sexually permissive behavior among Black females has racist and misogynist origins. However, participants described “fast” as the cultural term often used by Black women to label younger Black females in the Girl or Grown phases who looked or acted sexual. Participants recalled being called “fast” during their childhood. Some described internalizing this label and acting accordingly (i.e., engaging in sexual activities, dressing to “show off” their bodies, or having shame about their developed bodies). Participants in the Grown and Woman phases used “fast” to reclaim and own their sexual behaviors at various points in their lives. Kadijah, age 20, described Fast:

Fast basically means you’re just out here having sex with any and everybody… older people, they be like look at that ‘fast girl’ over there…you get called fast by talking to boys around your age, but you also get called fast by talking to an older person that you know you shouldn’t be talking too. You get called fast if you look a certain way…there’s like a lot of reasons you could be called fast at a girl age.

Participants described increased risk for STI as a major consequence of the Fast pathway. The Fast pathway was open to all three phases of sexual development. Those who identified with this pathway described themselves as growing up quickly and having a shortened childhood (i.e., Girl phase). Participants who were on the fast pathway often described themselves as entering the Grown phase as early as 11 years old.

#### Conditions influencing fast pathway

Conditions often leading participants on the Fast pathway included: (a) lacking protectors, (b) lacking information about sex, and (c) having traumatic life events. Participants described lacking protectors as not having adult guidance about sexual health matters. Most commonly, one or both parents were absent due to work schedules, inattention, incarceration, or death of parents or parental figure. Some reported not having a father figure. Some participants had been placed in foster care and had little protection or guidance during the Girl phase. Joseline, age 22, described how the loss of her father reduced her level of protection and contributed to her STI risk:

If I had a father around, I feel like I would probably still be a virgin. Before my father died, he was very strict. I feel like I wouldn’t have just ran free and did whatever…I probably wouldn’t have caught an STI…I would’ve not had as many relationships as I have had because I wouldn’t have needed to fill the void.

Inattentive parents were often described as mothers who were more interested in their boyfriends than their children or parents who abused drugs or alcohol. Some participants described using sex as a means to fill an emotional void and/or grieve the loss of a protective figure created by a missing or inattentive parent, particularly father figures. Consequently, they engaged in sex at early ages and had frequent sexual encounters. Sasha, age 27, described her lack of parental protection, “My parents being drug addicts, I had this idea about losing my virginity from a young age…because I was lacking that connection and that bond. I wanted to feel loved and wanted.” Dora, age 52, described how the loss of her parents influenced her choice for the Fast pathway, “My mom and dad, they died when I was a little baby. My grandmother and grandfather raised me, but they weren’t really there for me…that’s how I ended up with a STI…I wanted to be fast…I was a child.”

Participants reported lacking information about sex. Younger participants explained that their sex education during middle and high school was inadequate or involved scare tactics aiming to promote abstinence. Other teaching strategies focused on pregnancy prevention with little attention to STI. Joseline, age 22, stated, “Our school didn’t really have umm…Sex Ed class because most people there were already pregnant or had kids.” All participants described a “culture of silence” surrounding conversations about sex. They were discouraged from talking with adults about sexual health in their homes, school, and elsewhere within their communities. Even after receiving information from health care providers following STI diagnosis, participants often left appointments feeling confused and uninformed about STI prevention. Erica, age 20, described her experience with her health care provider, “I won’t go to a doctor, I don’t understand them…this is not making any sense, like what are they talking about?” She later described seeking sexual health information on her own and from friends. For participants, learning about sex occurred through their own personal experiences and experimentation, often leading them down the Fast pathway at some point in their development.

All participants described experiencing or witnessing a traumatic event (i.e., sexual abuse, rape, molestation, or physical abuse). Perpetrators were often family members, friends, or sexual partners. Ongoing sexual trauma was typically related to the lack of parental protection. Participants who experienced trauma often described themselves as engaging in risky sexual behavior well into their adulthood, typically leading to the Fast pathway:

You go down the Fast path…you are just trying to fill that void…I would definitely add the effects of not having a father in your life, the environment where you grew up in, your surroundings, your friends and also any traumatic events like losing somebody or being sexual abused or witnessing something happen, because I think that you’re trying to fill a void...It’s really the combination of all these things that can put someone on the fast path. (India, age 26)

#### Consequences of fast pathway

The two most commonly reported consequences the Fast pathway included: (a) early onset of sexual activity and (b) engagement in risky sexual behaviors. For those on the Fast pathway, first sexual encounters occurred between the ages of 10 and 16 years. Anika, age 34, attributed a sexual assault to her entry onto the fast pathway, “I was raped when I was younger…raped, molested, all that stuff…I want to say it was about ten when I consented to sex…So probably from…seventh, eighth grade to senior year,^3^ (I had sex with) maybe four guys…I was fast.” Such early sexual abuse and/or later being involved with partners who disregarded consent or ignored the girl’s young age may have predisposed some girls to the Fast pathway. Early pubertal onset also posed a threat to Black girls’ sexuality. In the absence of protectors, girls received unsolicited attention from older males and were sexually assaulted. These girls were often seen as sexually mature or capable of having sex because their bodies looked mature. Participants reported that, as girls, they often dated older Black men who were more experienced in sexual matters. These early sexual encounters increased the girls’ STI risk.

Participants described having unprotected sex, multiple sexual partners, and not knowing if their partners were having sex with others. Some participants talked about “showing off” their bodies (i.e., wearing little, short or tight clothing) to portray their sexuality or attract partners. Joseline, age 22, described acquiring multiple sexual partners as a competitive game, “I was sleeping with…probably five people, when I was about sixteen… It was like a competition.” Participants possessed little knowledge about how to protect themselves from STI and often relied on information from their peers. Latoya, age 19, described the naivety of her peers on the Fast pathway:

My friend is on the fast path…she is more sexual than I am. She is more ignorant in her sexual endeavors…I feel like there is pressure from peers to be fast anyone who come at her and shows her attention and affection, she throws herself at.

### Cautious pathway

Participants on the Cautious pathway took more precautions (i.e., used condoms, limited the number of partners, and had open conversations about sexual health)—all served to protect them from STI. Some described the Cautious pathway as a period of “slowing down”, increased self-reflection, and increased awareness of their sexual health. Latoya, age 19, described the Cautious pathway, “On the cautious path you are more selective and choosy about who you have sex with. You are going to wait and have sex with who you really want…you are in control and it is your decision.” Participants who identified with the Cautious pathway remained in the Girl phase longer and entered the Grown phase when they were older than did their peers on the Fast pathway. Those on the Cautious pathway tended to have protectors, were aware of sexual predators, and had the information they needed to make informed decisions about their sexual behavior.

#### Conditions influencing cautious pathway

Two prominent conditions influenced the choice of the Cautious pathway: (a) presence of protectors and (b) access to sexual health information. All participants on the Cautious pathway described the role of protectors in influencing their decisions about becoming sexually active. Protectors were physically available and provided guidance and support as parental figures. Although protectors were often mothers, sometimes they were other relatives or adult community members, who cared about the girl’s well-being and tried to keep them physically and psychologically safe. Latoya, age 19, described her older sister as her protector, “I definitely learned more about sexual health from her then I did from mom or dad…my sister took an interest in women’s health, made me realize how important my body is and taking care of myself.” Jennifer, age 42, explained how members of her community protected her:

The landlord that I got, he’s…Just like a father figure to me…I used to be on drugs. But he got me straightened out, I stopped sticking with the people that I used to and not letting people use me for my body.

Participants explained that when they were young, their protectors set limits on who they could befriend, where they could go, or how they dressed. Anika, age 34, described the lessons her parents taught her as a girl, “I was always taught keep your legs closed when you have on a skirt…you don’t want nobody looking in there.” Although the Cautious pathway was characterized by a strong presence of protectors during the Girl phase, such protection was not consistently available throughout their developmental process. Katrina, age 23, explained how her parents’ divorce disrupted her protection, “My mom and dad got divorced when I was like ten so I stopped living with my dad by then…he wasn’t there to protect me and my mom didn’t talk to me about my body, let alone sex.”

Participants also described the value of having health information to inform their decisions about sex. Such information was acquired actively or passively. Sources included the internet, doctors, trusted adults in their community, family members, friends, or formal college classes. Joseline, age 22, identified her physician, with whom she had a good relationship, as a valuable source, “I have a really cool doctor…my experience, was pretty good. I think a big part of it is how doctors deliver the news about the STI, it made me slow down.” After receiving health information, participants described feeling more confident and aware of their risks. Health information empowered them to enjoy sex while protecting themselves from STI, violence and abuse, unplanned pregnancy, and emotional harm. Some became protectors. Katrina, age 23, explained, “I’ll always offer condoms to my friends…getting an STI and finding out more information about it definitely helped me be more confident and, spread awareness to my friends.”

College courses in Gender and Women Studies (GWS) and African American History provided participants a historical perspective of Black female sexual oppression. These participants talked about how the historical violation of Black women’s reproductive rights (e.g., mass sterilization and rape during slavery) motivated them to take more proactive approaches to their own sexual health. Erica, age 20, described this:

Black women… historically we’ve been …disposable as in very easy to dismiss…dismiss your emotions, dismiss your feelings, dismiss when you inquire about something, dismiss when you are trying to be serious…it’s just like always brushed aside or thrown away…Now I am wanting to find holistic ways…for sexual health.

Participants also discussed the immense gender and racial discrimination that Black women face and how it has influenced their own perceptions of themselves and their sexuality. Increased awareness of their social conditions motivated participants to assert greater agency in their health care. Katrina, age 23, noted:

[It] wasn’t until my senior year of college when I took a GWS class that I got more of a scientific and historical view and just like the real details of contraceptives, STIs and pregnancy and how it effects women…I went home and taught myself more.

Information engendered a greater sense of empowerment, self-esteem, and pride in their sexuality. It gave them increased control over their own bodies and decisions about sex. Maya, age 19, “I have come to learn my self-worth and what I want and what I won’t do, which has put me more on this cautious path.”

#### Consequences of cautious pathway

Participants attributed two positive consequences to the Cautious pathway: (a) delayed age of initial sexual engagement and (b) protecting themselves. Participants on the Cautious pathway chose to delay having sex until they were older, found the “right” partner, and/or were more knowledgeable about sex. Participants commented that delaying sex until the age of 18 was uncommon within their social circles. Char, age19, explained, “I had sex when I was sixteen and that’s kind of older for my age…a lot of people are having sex before me.” Participants explained that waiting to find the right partner was connected to a sense of self-love, self-esteem, and self-worth. They also described wanting to be in control of their sexuality. They expressed that control by waiting and carefully choosing a partner who respected and valued them as much they respected and valued themselves. Angela, age 24, described her approach to being “cautious”:

I like getting to know my partner, making them get to know me and being accountable for the fact that they know me as a person before they see me as something sexual…they see that I am a person first. That is me protecting myself…because people then decide if they want to make that type of investment in me…And I choose if I want to engage in sex.

Protection was described as physical and emotional. Physical protection involved use of barriers (i.e., condoms) during sex and birth control. Latoya, age 19, said, “[I] realize how important my body is and taking care of myself. She [my sister] encouraged me to get this IUD because I am not ready for a child.” Emotional protection was described as nurturing self-respect and requiring partners to agree to a monogamous sexual relationship. After being in a relationship that led to an STI, Mariah, age 26, explained, “I would say I protect myself emotionally…there is an emotional component to sex…I am going to limit who I have sex with because I just emotionally can’t handle it.”

### Conditions influencing movement from fast to cautious pathway

All participants described their own bidirectional movement between the Fast and Cautious pathways. Movement between the pathways can occur at any time/phase during the developmental process. Conditions that prompted participants to shift from the Fast to the Cautious pathway included (a) life events (i.e., STI, entering serious relationships, having children to protect) and (b) supportive relationships. The most commonly discussed event that caused such a shift was an STI because it signaled a need for self-protection and different choices about partners. India, age 26, described her shock after contracting an STI, “Getting the STI was the one moment where I was like whoa. I need to slow down because I could not pinpoint where I got it from.” Some women learned vicariously from the mistakes of their friends, who did not change their behavior upon contracting STI. Latoya, age19, explained:

I would hope an STI would make that shift to being more cautious, but I have seen examples in which it has not. They have made no type of change. Like they’ve had scares, but they just continue down the Fast path.

Jennifer, age 42, became more cautious after she contracted an STI from an unfaithful partner, “I like him…But, I know he probably is out here messing around with other women, now since I got this disease [STI]. So that’s telling me now, it’s time to use condoms with him.” By contrast, participants on the Cautious pathway described using condoms, limiting the number of partners, having candid conversations with partners about sexual health, and getting to know potential partners before having sex. Participants described other life events (e.g., an abusive relationship) as reasons to become more cautious. Felicia, a 40-year-old divorced participant, explained how being in an abusive marriage led to her become cautious in subsequent relationships, “I’m not going to fight my husband to be with my husband. I had to walk away…He stalked me… He tried to rape me… It caused me to be cautious.”

For some, having a child was the life event that led them to reconsider their choices and switch to the Cautious pathway. Becoming a parent roused a sense of protectiveness for their child and a need to set a good example. Having a child was a responsibility that required prioritizing the child’s care over their own sexual needs or desires. Dora, age 52, described her transition from being Fast, “I was doing me, I was having fun and look what I end up with, two STIs and a baby when I was seventeen…now it’s time for me to be this woman that I’m supposed to be.”

Participants also described how supportive relationships helped them shift from the Fast to Cautious pathway. Supportive relationships offered them health information and affirmation of their value as Black women who were more than sexual objects. Maya, age 19, stated, “By surrounding myself with people who are going through the same thing… getting more information…constantly reminding myself … you’re not just a sexual object”. Supportive sexual partners prompted the shift by helping participants feel secure in mutually respectful relationships. Ada, age 26, stated, “I consider my partner to be my protector. I feel like I am able enough to protect myself and I feel like this is where I am currently in my sexual journey.” It is noteworthy that one participant described an inability to move from the Fast to Cautious pathways. She attributed her struggle to her history of repeated traumas, multiple STI, and lack of protection. Brandy, age 23, explained:

I got put out of my mom’s house for being fast…I never had that man figure in my life…I got touched by my cousin when I was little…then I got an STD from my boyfriend cheating… I went down this path of just sleeping with people because I didn’t really care because my first boyfriend was a jerk…then this guy tried to assault me, but I begged him to leave…there was no one there to protect me…I felt so defenseless.

Participants gained information in a variety of ways. Supportive relationships and college courses often afforded participants access to health information to keep them safe from STI. For example, discovering the consequences of others’ behaviors often prompted participants to change their own behaviors and become more cautious.

### Conditions influencing movement from cautious to fast pathway

These conditions included: (a) changing environments (i.e., college), (b) substance use, and (c) peer pressure. For some, leaving the protection of their homes and parental oversight, particularly mothers, to new environments precipitated pathway shifts. Such shifts often occurred during the Grown phase. Moving to live on a college campus was a prime example of changing environments, where parental rules and boundaries no longer existed. Participants made their own decisions, including sexual behaviors. Several participants stated that when they left home, they “forgot” the values that their parents instilled in them. Such values included waiting until marriage to have sex and not focusing on boys or sex. Sheila, age 57, described her experience in college:

My momma always talked about don’t do it. Stay a virgin. And so, I tried to stay, true to that. But when I got in college my momma wasn’t there…You’re grown…and you’re in a relationship, you are so in love. You want that to work and sometimes you give up your values when you know… that’s not the best decision to have sex unprotected.

Other participants echoed these sentiments about college becoming a time of experimentation with sex, drugs, and alcohol. Use of recreational drugs and alcohol often led to unprotected sex. Additionally, having multiple partners was acceptable because “everyone [within their peer group] was having sex”. Ada, age 26, explained, “College is like the accepted Fast stage, like everybody in college is a hoe.” Katrina, age 23, shared her experiences with alcohol, “As a freshman, you start drinking like right away…alcohol is just, a terrible mixture…It just makes you not think clearly….and do things like…have sex or you make decisions that you normally wouldn’t.” Freshman refers to a first-year student of any gender at a university, college, or high school.

Peer pressure also shifted participants from Cautious to Fast, particularly during the Grown phase. Peers included friends, acquaintances, and sexual partners. Kadijah, age 20, stated, “Your friends like oh, you should try it (sex), or you’ll just see somebody acting like oh, okay, like she’s doing it, then okay then I’m going to do it too.” Char, age 19, noted that, “Guys always peer pressure a girl into doing something sexual, or maybe a girl could peer pressure a guy into doing something.” Peer pressure in combination with the new environment, influence of drugs and alcohol, and absence of protectors was almost a prescription for the Fast pathway. Latoya, age 20, explained:

You choose the fast path and go with who is giving you the most attention because you didn’t have parents and you want that attention. Or you feel like having sex is what you should do because of the outside factors peer pressure, society that is on you.

In short, movement between the two pathways was influenced by a variety of sociocultural conditions such as, sexual health knowledge, STI, personal choice, sexual trauma history, and presence or absence of protectors (see Table 1).

**Table 1:** Identified sociocultural conditions

Category	Frequency	Examples
**Fast pathway**	17	“Fast basically means you’re just out here having sex with any and everybody… older people, they be like look at that ‘fast girl’ over there…you get called fast by talking to boys around your age, but you also get called fast by talking to an older person that you know you shouldn’t be talking too. You get called fast if you look a certain way”
**Conditions influencing the fast pathway**		
Lacking protection	11	“If I had a father around, I feel like I would probably still be a virgin. Before my father died, he was very strict. I feel like I wouldn’t have just ran free and did whatever…I probably wouldn’t have caught an STI”
Lacking information about sex	10	“Our school didn’t really have umm…Sex Ed class because most people there were already pregnant or had kids”
Traumatic life events	11	“Traumatic events like losing somebody or being sexual abused or witnessing something happen, because I think that you’re trying to fill a void…it’s really the combination of all these things that can put someone on the fast path”
**Consequences of the fast pathway**		
Early sexual engagement (< 16 years old)	11	“I was raped when I was younger…raped, molested, all that stuff…I want to say it was about ten when I consented to sex…So probably from…seventh, eighth grade to senior year, (I had sex with) maybe four guys…I was fast”
Engagement in risky sexual behaviors	14	“I was sleeping with…probably five people, when I was about sixteen… It was like a competition.”
**Cautious pathway**	18	“On the cautious path you are more selective and choosy about who you have sex with. You are going to wait and have sex with who you really want…you are in control and it is your decision.”
**Conditions influencing the cautious pathway**		
Presence of protection	10	“I definitely learned more about sexual health from [my sister] then I did from mom or dad…my sister took an interest in women’s health, made me realize how important my body is and taking care of myself”
Having sexual health information	9	“I have a really cool doctor…my experience, was pretty good. I think a big part of it is how doctors deliver the news about the STI, it made me slow down.”
**Consequences of the cautious pathway**		
Delayed age of initial sexual engagement	11	“I had sex when I was sixteen and that’s kind of older for my age…a lot of people are having sex before me.”
Protecting their own bodies	18	“I like getting to know my partner, making them get to know me and being accountable for the fact that they know me as a person before they see me as something sexual…they see that I am a person first. That is me protecting myself”
**Conditions influencing movement between fast and cautious pathways**		
Major life events	18	“I like him…But, I know he probably is out here messing around, now since I got this disease (STI). So that’s telling me now, it’s time to use condoms with him.”
Needing to protect others	11	“If you have kids…it is your responsibility to protect”
Supportive peers	4	“By surrounding myself with people who are going through the same thing… getting more information…constantly reminding myself … you’re not just a sexual object”
**Conditions that prompted a shift from the cautious to the fast pathway**		
Changing environments	9	“College is like the accepted Fast stage, like everybody in college is a hoe.”
Influence of substance use	10	“As a freshman, you start drinking like right away…alcohol is just, a terrible mixture…It just makes you not think clearly….and do things like…have sex or you make decisions that you normally wouldn’t.”
Peer pressure	13	“Guys always peer pressure a girl into doing something sexual”

*Early sexual engagement was defined as having sex under the age of 16 supported by CDC and Heywood, W., Patrick, K., Smith, A. M., & Pitts, M. K. (2015). Associations between early first sexual intercourse and later sexual and reproductive outcomes: a systematic review of population-based data. *Archives of sexual behavior*, *44*(3), 531–569

## Discussion

Our findings expand knowledge regarding the sexual development of Black females. The conceptual model explains critical sociocultural conditions influencing sexual risk throughout the life course. The two sexual pathways (Fast and Cautious), not previously identified in the literature, are grounded in the sociocultural context and sexual experiences of Black women. Our findings explain how certain social circumstances, systems, community-wide hardships, and cultural expectations facilitate or impede choice of pathway and their consequences to the sexual development and health of Black females. Lack of sexual health information, experience of traumatic events, the psychological impact of absent parental figures make movement to the Fast pathway more likely, and leaving it more difficult. Presence of caring adult protectors and access to sexual health information help girls and women stay on the Cautious pathway and potentially mitigating STI risk. These findings point to potential opportunities to support the development of Black female sexuality in ways that respect their autonomy in making choices about the Fast and Cautious pathways, while protecting their reproductive health, particularity related to STI. This conceptual model can inform the future study of protective interventions for Black females at various junctures in their sexual development across the life course.

Findings highlighted the important role of protectors is consistent with other researchers who have identified the family structure (i.e., familial values, roles, and expectations) as well as parental communication, monitoring, discipline, and authority as protective factors relating to STI risk in adolescents [56–58]. Research has shown that adolescents’ who report close relationships with their parents are less likely to initiate sexual intercourse at an early age [59, 60]. A study of 212 adolescents’ attitudes regarding sexual behavior found that young people who communicate with their parents about sexual issues are more likely to delay sexual debut [61]. Protective family processes (i.e., close satisfying relationships with parents, communicating about risk behavior, and having clear parental norms about behavior) discourage risky sexual behavior among Black adolescents during their transition to adulthood [62]. Another study found that two parent families, open family communication, and social autonomy encourage a delay in sexual onset [63]. Similarly, our findings showed that protectors influenced Black girls’ choices of the Cautious pathway and mitigated threats to the sexual health of vulnerable Black girls.

The relationship between grief due to the loss of a paternal figure and becoming sexually active at a young age deserves further investigation. We need a greater understanding of how the grief response in combination with normative sexual curiosity, hormonally driven urges, and social messaging interact to influence girls’ choices about sex. This issue may be particularly pertinent to Black girls who have lost a male family member due to death or incarceration. Our findings highlight the need to unpack the grief, trauma, and shame discourse around Black female sexuality and normalize the autonomy of sexual pleasure and desire at all phases of sexual development.

Our findings suggest that having health information and effective communication about sexual health (i.e., found among college-educated participants) influenced participants’ decisions about engaging in sexual activity. Paradoxically, college served as a condition for increased sexual activity due to campus norms and more self-protective behaviors due to access to health information. Research has shown a relationship between socioeconomic status, often conferred by higher education, and sexual health. In a study of 766 female college students, participants expressed a desire to have conversations with their parents about sex, delivered in a non-judgmental manner [12]. Such information could equip college-aged females with tools to make informed decisions about sex. Chandler et al. [12] also found that peer support may be most influential regarding sexual behavior during college because college students are no longer under constant surveillance by their parents. These collective findings highlight the influence of peer support. Thus, peer led prevention programming may be important in reducing STI risk.

Our results suggest that Black girls are particularly vulnerable to sexual engagement when they develop early sexual characteristics. Black girls tend to experience menarche at a younger age than their white counterparts [64]. Previous studies have found that girls who mature early are more likely to engage in sexual intercourse and Black girls report sex before the age of 13 [65]. Our findings support these results as participants in this study reported early sexual development and engagement. Some research suggests that these racial differences may be explained by socialization patterns or cultural expectations. The relationship between early pubertal development and psychosexual behaviors in Black girls merits further investigation.

The concept of consent or assent by Black girls under the age of 18 needs to be further examined within Black communities. Participants explained that because they looked older than they were, men often viewed them as being able to consent to sex. However, physical development does not necessarily correspond with psychological maturity. In the US, legally, minors (< 18 years) are not considered capable of making decisions about important life choices (i.e., alcohol use, marriage, medical procedures, and sexual intercourse). As our findings show, minor children rely on their protectors to look out for their best interests. Additionally, some participants retrospectively described “choosing” pathways which suggests they took responsibility, ownership, and in some circumstances blame for what, at the time, might actually have been highly constrained options or consensual choices. Gaining a greater understanding of the meaning of “healthy sexual choices” at the various phases of sexual development could inform interventions designed to prevent Black girls and women from engaging in risky sexual behaviors. Research also needs to examine conceptions of “consent” within the context of sex within Black communities.

Although sexual trauma was not the focus of our study, participants reported such experiences. Our findings echo research showing disproportionate rates of sexual abuse and violence perpetrated against ethnic minority women, specifically Black girls and women [3, 66]. A study with 290 women raised in two-parent homes, found a higher prevalence of childhood sexual abuse among Black women (34.1% [67]) compared to white women (22.8% [35]). Protective factors included family structure (i.e., living with two biological parents throughout childhood) and higher social class [66]. Our findings add to growing evidence of society failing to protect Black females across the life course [10, 14, 17]. Additionally, participants in our study described “filling the void” of a missing father figure with sex. Future research could utilize the life course perspective, attachment theories, and trauma-informed care as frameworks to examine relationships between childhood sexual trauma or loss and adult sexual relationships. Even girls with sexual health information, good parental support, engaged in risky behaviors and choose the Fast pathway. Therefore, evidence-based interventions are needed to protect Black girls and women at all phases of sexual development.

### Implications

Findings point to four windows of opportunity for interventions at various levels (family and community, health care, research, and policy) to improve the protection of the sexual and reproductive health of Black girls and women. First, it is of paramount importance that families and communities address the generations of sexual trauma in Black communities and unpack the historical and sociocultural complexities that pose threats to Black female sexual health. Participants on the “Fast” pathway did not have protectors in their lives. Their mothers or caregivers likely also lacked such protection themselves. Participants' use of the term, “fast”, to describe themselves or other members of their community underscores the detrimental legacy of Black female oppression. Racist and misogynist societal factors that allow such experiences to remain “normative” for so many Black girls must be eradicated. Additionally, internalization of this type of oppressive language and self-referencing among Black women and girls may negatively affect their mental health (e.g., self-esteem, self-concept). Future interventions might include helping families engage in discussions about culturally embedded norms (i.e., sex at young age or sex with older men) and use of stigma-laden language (i.e., Fast) to help Black girls make healthy sexual choices. More attention also needs to be paid to developing community-based, culturally safe, psychosocial interventions to support Black girls and women in grieving loss, healing from emotional trauma, as well as fostering intrapersonal growth and self-actualization.

Second, health care providers must ensure Black girls have access to health care information and services regardless of which sexual pathway they choose. Various sociocultural protective strategies (i.e., physical, verbal, cultural) have been used by Black women to protect Black female sexuality [17]. Health care providers should assess the availability of protectors to young Black girls, provide culturally congruent sexual health information, guide them in decisions about sex, and teach them how to develop mutually respectful intimate relationships. Such health interventions may also be embedded in the school, implemented by school nurses or counselors as school may be a particularly vulnerable setting for Black girls. Measures to assess risk based on sociocultural conditions including lack of protectors may be critical in health care and school settings. As protectors shift and are often absent after the Girl phase, both the Girl and Grown phases (ages 11–18) may be an optimal time for sexual and reproductive health programming. This programming could include instilling self-protective strategies (i.e., communication about consent or safer sex practices) among Black girls.

Third, researchers need to develop and evaluate STI/HIV prevention interventions designed to equip Black girls and women with skills in making healthy sexual choices. One might expect that having contracted STI would prompt a shift from the Fast to Cautious pathway, however, that was not always the case. A greater understanding of the barriers that prevent some Black girls and women from changing their sexual behavior when they want to change, but feel stuck and are unable to do so is needed. Research should include a more sex-positive focus on Black female sexuality. Further examination of the use of “fast” is necessary to determine how this is associated with self-esteem, self-control, and mental health [34]. More research is needed on protective factors and the Cautious pathway can help girls learn how to make agentic decisions about their sexuality, recognize their own inherent value, and become assertive self-advocates within the context of their sexual relationship. Our study found a relationship between the absence of a father figure and the Fast pathway, as their absences often led Black girls to engage in risky sexual behavior. Understanding other mediating or moderating factors can inform interventions that focus on family communication and parent-daughter relationships that support their efforts to protect Black girls’ sexual development and prevent sexual exploitation. Our findings also demonstrate that Black male protectors can take various forms beyond the role of fathers (i.e., landlords, neighbors, brothers, cousins, teachers, couches), and community-based interventions could be broadened to include Black men in these various capacities. Additionally, the role of Black men in Black female sexual development needs to be further explored.

Fourth, systemic disruption in family units was found to be an important factor influencing the lack of protection among Black girls. It is well established that unemployment and incarceration often prevent Black men from fulfilling their roles as fathers and protectors [30]. Thus, it is essential to ameliorate other forms of institutionalized racism and discrimination so that Black men can remain engaged in the lives of their families. In addition to people serving as protectors, policymakers need to look at the systems, laws, and structures that prevent the protection of Black girls. An example of this may include examining custodial guardianship policies and stipulations around Black men being involved in their children’s lives. Another example may include surveying or further examining systems that impede Black men’s ability to engage in the protection of Black girls.

### Limitations

Although our inclusion and exclusion criteria did not preclude participants who identified as lesbian, bisexual, or queer, most self-identified as heterosexual. Therefore, the transferability of our findings may be limited to Black heterosexual girls and women in the US. This study did not include demographics related to the racial composition of participants’ neighborhoods environment or direct inquiry about sexual concurrency, which might have offered additional insights about conditions that shaped participants’ sexual experiences. Additional information about the type of sexual partnerships they engage in and the sexual education participants received should be collected in future studies. Initially, we recruited only women who had a history of STI; therefore, 75% of the participants had experienced STI which may not be representative of the general Black female population. The sample was restricted to Black females over the age of 18 years, who retrospectively described their sexual experience of childhood and adolescence. Therefore, prospective studies of Black female adolescent psychosexual development are needed to verify our findings.

## Conclusion

Black girls and women face unique sociocultural conditions that increase their risk for STI and impact their sexual development. Our findings extend the literature by producing a conceptual framework grounded in their voices and experiences to explain the Black female sexual developmental processes and conditions that affect their choice of pathways across the life course. This model can be used by scholars, researchers, and practitioners concerned with understanding the intersectional experiences, sexual health, and sexual behaviors of Black females in the US. Findings point to a need for future research to develop multi-level interventions focusing on specific phases of Black female sexual development.

## Data Availability

The datasets generated and/or analyzed during the current study are not publicly available due to the nature of qualitative data as these may contain information that could compromise participant identity but are available from the corresponding author on reasonable request.

## Footnotes

1 Grown is a term commonly used in Black American communities. The sample, described Grown as the second phase in the sexual development of Black females. However, sometimes those in the Girl phase “behaved” as if they were Grown when they were not [19].

2 The GED is a high school equivalency diploma in the US, which can be used to apply to college or for a job similar to a high school diploma. The GED test can be completed by those who have completed the 12th grade.

3 Children are typically between 12–13 years old in seventh or eighth grade in the US educational system.
